# Supplementary figures and images for: Distribution and ecological segregation on regional and microgeographic scales of the diploid Centaurea aspera L., the tetraploid C. seridis L., and their triploid hybrids (Compositae)
Source: PeerJ. 2018 Jul 3;6:e5209. doi: 10.7717/peerj.5209 (PMC6034602; doi:10.7717/peerj.5209)

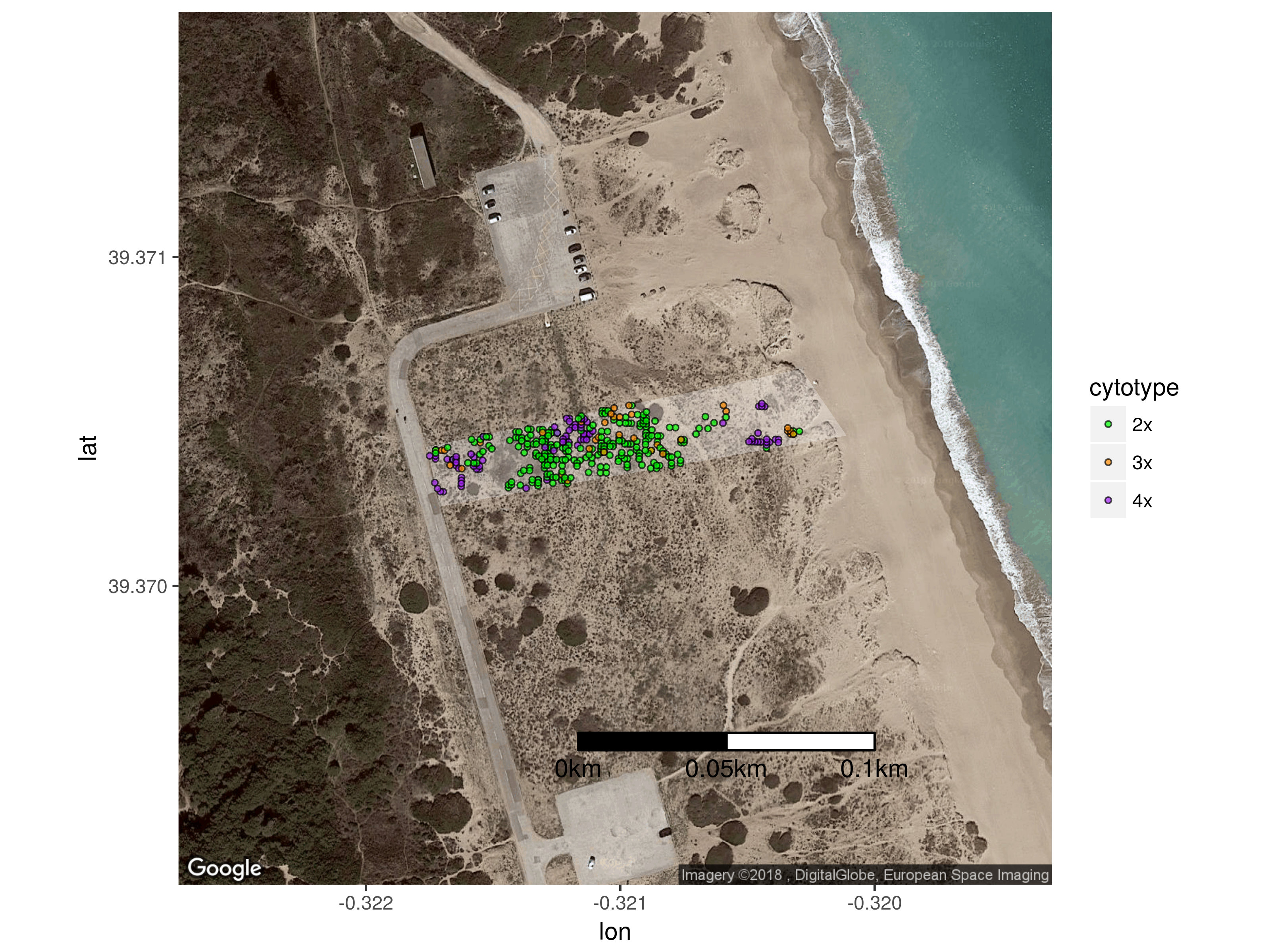

Supplement: Supplemental Information 2 — Diploid individuals of C. aspera are represented by green circles, tetraploid individuals of C. seridis by purple circles and triploid individuals of C. x subdecurrens by orange circles. Orthophotograph by Imagery ©2018 DigitalGlobe, European Space Imaging. [file peerj-06-5209-s002.png]

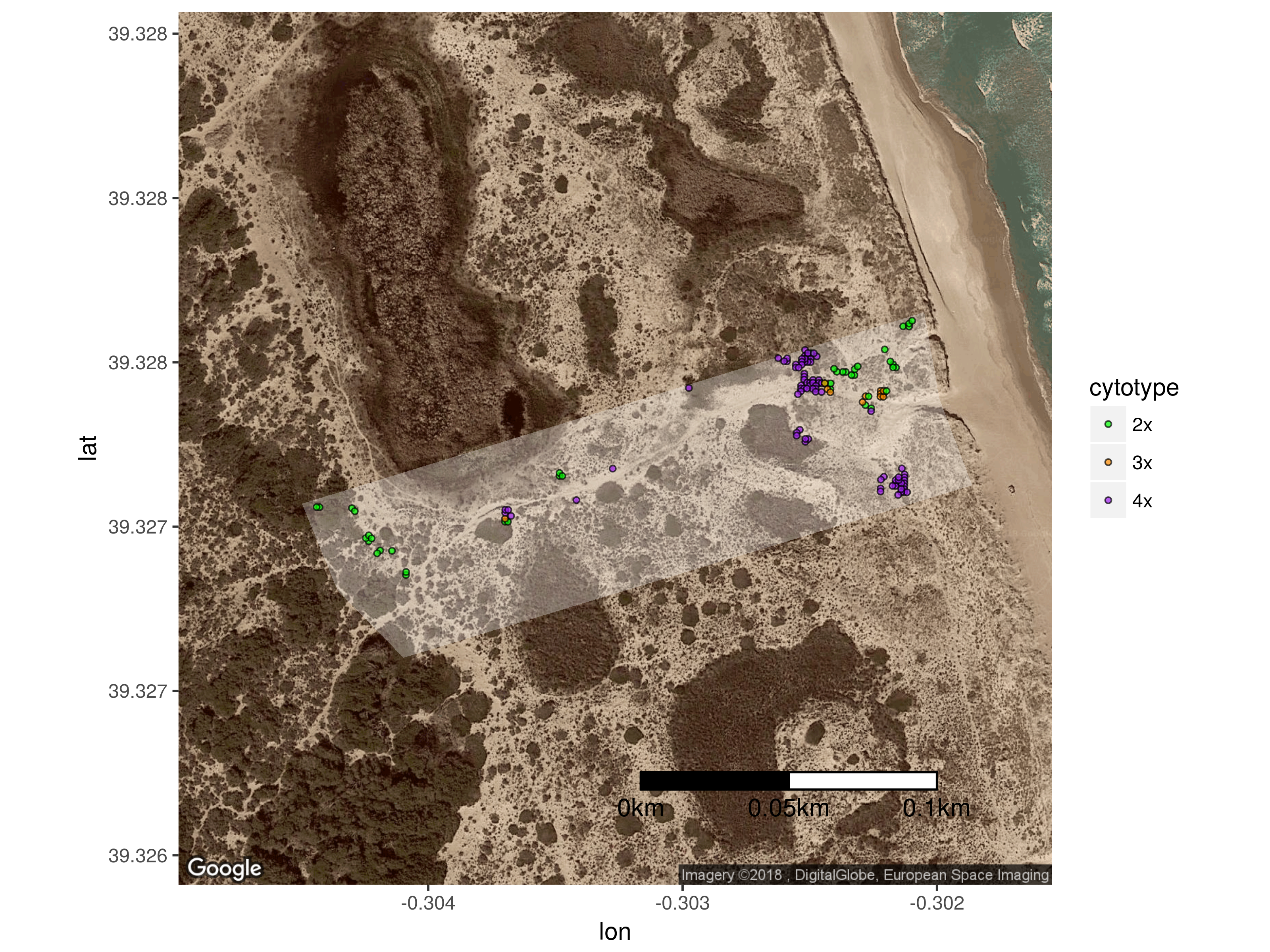

Supplement: Supplemental Information 3 — Diploid individuals of C. aspera are represented by green circles, tetraploid individuals of C. seridis by purple circles and triploid individuals of C. x subdecurrens by orange circles. Orthophotograph by Imagery ©2018 DigitalGlobe, European Space Imaging. [file peerj-06-5209-s003.png]

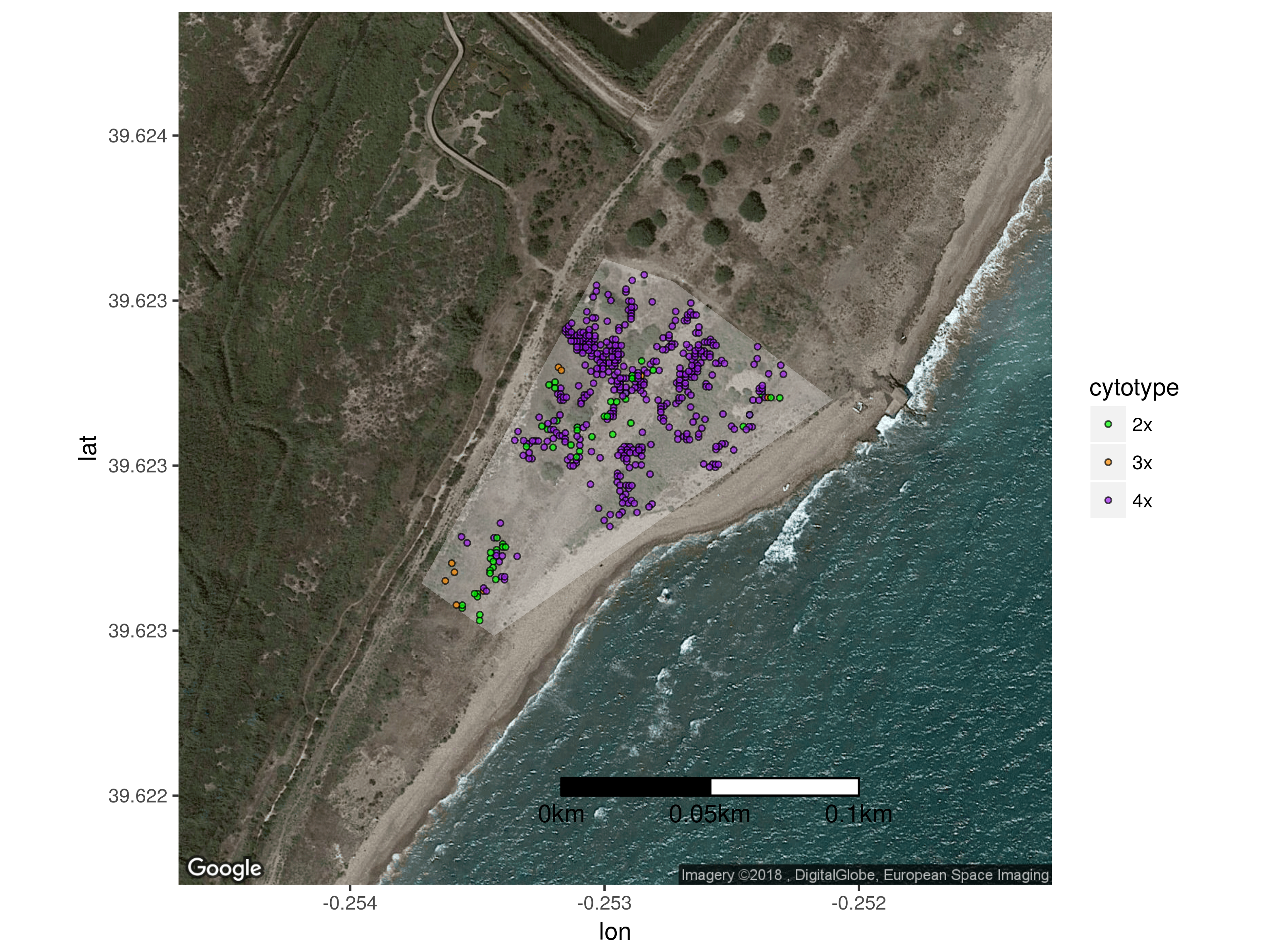

Supplement: Supplemental Information 4 — Diploid individuals of C. aspera are represented by green circles, tetraploid individuals of C. seridis by purple circles and triploid individuals of C. x subdecurrens by orange circles. Orthophotograph by Imagery ©2018 DigitalGlobe, European Space Imaging. [file peerj-06-5209-s004.png]
